# The direct cost incurred by patients and caregivers in diagnosing and managing prostate cancer in Ghana

**DOI:** 10.1186/s12913-022-08476-3

**Published:** 2022-08-31

**Authors:** Ebenezer Wiafe, Kofi Boamah Mensah, Kwaku Addai Arhin Appiah, Frasia Oosthuizen, Varsha Bangalee

**Affiliations:** 1 Discipline of Pharmaceutical Sciences, College of Health Sciences, University of KwaZulu-Natal, Durban, South Africa; 2 Clinical Pharmacy Services Unit, Directorate of Pharmacy, Ho Teaching Hospital, Ho, Ghana; 3 Department of Pharmacy Practice, Faculty of Pharmacy and Pharmaceutical Sciences, College of Health Sciences, Kwame Nkrumah University of Science and Technology, Kumasi, Ghana; 4 Department of Surgery, School of Medicine and Dentistry, College of Health Sciences, Kwame Nkrumah University of Science and Technology, Kumasi, Ghana

**Keywords:** Cancer financing, Cost-of-illness, Pharmacoeconomics, Prostate cancer, Ghana

## Abstract

**Background:**

Over the years, the prevalence of prostate cancer (PCa) has been on the increase. Poor prognosis has been a reflection of increased advance-staged diagnosis and inadequate financial assistance. The prioritization of resources cannot be effective enough to factor in the unexpected economic burden resulting from ill health unless health economic approaches are utilized to estimate the cost of diseases including PCa. With the absence of data on the cost of PCa in Ghana, and the evidence of the benefits of PCa cost-of-illness studies on cancer financing, it has become imperative to investigate the direct health cost of PCa on patients and careers. Hence, we investigate the cost of PCa diagnosis and management, the availability and prices of PCa medications, and the affordability of PCa care in Ghana.

**Methods:**

The prevalence approach to cost-of-illness studies was adopted in this study through a random selection of two (2) hospitals, four (4) private laboratories, and ten (10) private community pharmacies in the Ashanti Region of Ghana. The diagnostic and management cost of PCa was investigated through the application of validated data collection instruments to representatives of the selected hospitals and laboratories. The availability and prices of PCa medications were studied with the administration of a validated tool to representatives of the selected pharmacies. The data were analyzed with Microsoft Excel Spreadsheet and the affordability of care was assessed considering the 2021 Ghana National Daily Minimum Wage (GNDMW).

**Results:**

The cost of diagnosing non-metastatic and metastatic PCa were respectively estimated at GHC 1686.00 ($ 290.58) and GHC 6876.00 ($ 1185.09). Radical prostatectomy, as a management option, was estimated at GHC 2150.00 ($ 370.56) higher than Extended Beam Radiotherapy (GHC 2150.00: $ 370.56). The mean PCa drug availability for the sampled pharmacies around the public hospital, all the sampled pharmacies, and around the private hospital were respectively 61.54, 51.54, and 41.54%. None of the sampled drugs at the stated strengths had a 100% availability. A 6-month androgen deprivation therapy employing goserelin was GHC 3000.00 ($ 517.05). The median drug price ratio (MDPR) was 0.72 - 15.38, with generic bicalutamide 150 mg tablets as the cheapest and generic flutamide 250 mg tablets as the most expensive.

**Conclusion:**

The diagnostic and management cost of PCa currently overwhelms the average Ghanaian because the minimum daily wage in 2021 is GHC 12.53 ($ 0.46). A higher economic burden was associated with metastatic PCa and hence, the need for strategies to improve early detection. Also, the inclusion of PCa management in the National Health Insurance Scheme would lessen the financial burden of the disease on patients and careers, and improve management outcomes.

**Supplementary Information:**

The online version contains supplementary material available at 10.1186/s12913-022-08476-3.

## Background

According to the Global Cancer Incidence, Mortality and Prevalence (GLOBOCAN) 2020 report, prostate cancer (PCa) (14.1%) ranks next to lung cancer (14.3%) amongst cancers affecting men. GLOBOCAN also estimated a 20% chance that every individual could develop cancer in a lifetime, whilst mortality in men was determined to be 12.5% [1]. In Africa, the late diagnosis of PCa has been a health concern as evidence shows that 64% of PCa patients died within 2 years of diagnosis at terminal stages [2]. An earlier mortality study in Ghana at the Korle-Bu Teaching Hospital (KBTH) reported PCa (17.35%) as second to liver cancer (21.15%) in men [3]. Recent data from the Kumasi Cancer Registry, 2015, indicate a rise in the prevalence rate of PCa amongst Ghanaian men where PCa was second in 2012 and first in 2015, and the fourth commonest cancer (9%) in both sexes [4].

The late detection of PCa in Ghana has been a public health issue [5] as the stage at diagnosis is a reflection of cost and prognosis [6, 7]. Data on the stage-specific cost of PCa varies and is also not readily available in the Ghanaian system. This situation leaves the newly diagnosed PCa patient confused as he does not know the financial cost required of him or his caretakers. The evidence from Ghanaian epidemiologic studies indicates that PCa is usually diagnosed at advanced stages [5, 8]. An awareness or knowledge of the cost of diagnosing and managing the disease could influence men to screen and if possible, advocate for early detection. In other countries where there is data availability, the direct health cost of PCa has successfully been studied and estimated [9, 10].

Cost-of-illness (COI) studies have been used in estimating and evaluating the economic burden of chronic medical conditions such as PCa [6, 11]. The concept of COI studies considers the effect of the disease being studied, on the health parameters pertinent to the geographical location of the study. Also, the particular region(s) of the country of the study, the communities, and the group of individuals usually affected by the disease are considered [12].

With evidence of women playing a vital role in PCa control in our previous review study [13], the cost of diagnosis and management of the disease is essential for women to prioritize their family resources and how the country would allocate its national resources to control PCa. The evaluation of the COI of PCa, through an analysis of the direct health cost due to the unavailability of data, in Ghana is therefore essential. Hence, this study seeks to determine the direct health cost of PCa diagnosis and management in Ghana. In achieving the set aim, we put forward the following specific objectives:

1. To determine the cost of diagnosis.
2. To conduct a cost analysis into the stage-specific management of prostate cancer according to local management protocols.

To present to the scientific community, the situation of PCa medications in Ghana, we assess the availability, prices, and affordability of pharmacotherapeutic agents used in the management of prostate cancer.

## Methods

The methodological approach adopted in this study was the prevalence approach because the researchers were interested in the stage-specific cost of PCa diagnosis and management [11]. Due to the absence of a national PCa care financing agency in Ghana and the lack of a universal PCa management cost sheet, we sourced data from health facilities including a public hospital, a private hospital, community pharmacies, and laboratories. The adopted prevalence approach was modified regarding the data sources, nonetheless, the method adequately analyzed and assessed the direct health cost from the payer’s perspective [11]. Additionally, the method sufficiently defined the disease condition, the geographical location of the study, the community, and the population concerned [12].

### Study settings, instruments, and subjects

The study was conducted in the Ashanti Region of Ghana because the region is endowed with a major public cancer management centre that serves several regions in the country. The study sites include a public hospital, a private hospital, ten (10) private community pharmacies, and four (4) private laboratories. Three (3) data collection instruments were employed in the study: Additional files 1, 2 and 3. Additional file 1: The diagnostic cost sheet, which focused on the cost components of the various laboratory workouts in the diagnosis of PCa; Additional file 2: The stage-specific management cost sheet, focused on the cost of the available management approaches; and Additional file 3: The availability and prices of PCa drugs, focused on the availability and the prices of selected PCa management medications. The participants of the study were recruited from these sixteen (16) facilities. The participants responded to the questionnaires after the study had been explained, and informed consent was duly obtained.

#### Inclusion and exclusion criteria

Only one public hospital was selected in the Ashanti Region because that is the only referral centre and one of the two national cancer management centres. The researchers selected a private hospital known to provide PCa management services. This strategy was to get a representation for the public and private hospitals involved in the management of PCa. Five (5) private community pharmacies within a 100-m radius of each hospital were randomly selected. This is because, from the researcher’s experience, these community pharmacies are within walking distance from the included hospital, and patients and caregivers will explore the availability of their prescriptions within the stated proximity. The participants recommended private laboratories that provide diagnostic support to their PCa patients. For uniformity, 5 private community pharmacies and 2 private laboratories were selected per hospital. According to ethical principles, facilities that did not consent to the study were excluded.

### Direct health cost estimates

The data on the direct health cost of PCa management was collected using three (3) data extraction instruments: Additional files 1, 2 and 3. These validated instruments are available on request [14, 15].

#### Determination of the cost of diagnosis

According to Wong and Bradley, patient registration is an integral facet of healthcare as it provides an avenue for healthcare facilities to keep adequate medical records on patients [16]. In Ghana, this service would incur a direct cost on PCa patients because the National Health Insurance Scheme does not pay for the management of the disease. Therefore, we classified the cost of patient registration, consultation, and other fees paid at the point of facility entry as patient entry costs: Additional file 1. Data on this cost component was collected from the hospitals and private laboratories by the Principal Investigator (PI). However, the private laboratories were restricted to consultation fees because some private laboratories are known to bill patients for specific consultancy services. The community pharmacies were not considered in estimating the entry cost because they provide consultancy, drug information, and other pharmacist-centred services to patients without charges.

The itemized cost of the laboratory investigations: prostate-specific antigen (PSA), free PSA, transrectal ultrasound (TRUS), and biopsy conducted for patients who reported at the hospitals with an impression of prostate and prostate-related conditions were investigated to confirm their availability and cost through the application of Additional file 1. Other investigations such as liver function test (LFT), renal function test (RFT) including serum electrolyte assessment, full blood count determination (FBC), blood group determination, serum phosphatases (acid/alkaline) assessment, computed tomography (CT) scan, magnetic resonance imaging/scan (MRI) - Whole Body Diffusion, and chest radiograph (X-ray) constituted the third modular cost items as indicated in Additional file 1 and complemented clinical management decision making. The PI investigated selected PCa patients’ list of private laboratories or diagnostic/imaging establishments where they seek their services. The PI randomly selected and made contact with 2 private laboratories or diagnostic/imaging establishments per hospital to investigate the availability and cost of the modular items.

#### Determination of the stage-specific management approaches and cost

The representatives of the hospitals responded to the data collection tool regarding the stage-specific management options and their costs: Additional file 2. The tool had been designed to determine the management approaches according to the invasiveness of the malignancy [17], the Gleason grading system [18, 19], and the Tumour, Node, Metastasis (TNM) staging system [20]. The various pharmaceutical agents, excluding adjuvants and other non-oncologic agents, that were employed in the management of PCa patients were listed by the hospitals’ representatives. The PI generated a master drug list used in the next stage of the COI study - the availability and price of pharmaceuticals.

#### Assessment of the availability and prices of pharmaceuticals

To report on the supply chain status of PCa medications in Ghana, we studied the availability profile and the prices of these medicines. Five (5) community pharmacies each were randomly selected from the vicinities, within walking distance, of the public and private hospitals and Additional file 3 was administered to their representatives. The PI sent the drug list to the pharmacists of these selected pharmacies to obtain information about the availability and prices of these drugs. The drug list document requested the community pharmacists to provide information on the makeup of the drugs (originator brand or generic), the available strengths of the medications, the dosage forms/units, the pack sizes, and the unit prices.

### Analytic strategies

The median itemized cost was calculated and used to estimate the direct PCa diagnostic and management cost [21] and the prices of pharmaceuticals used to manage PCa using a Microsoft Excel Spreadsheet [22]. The outcomes of the median cost assessment were presented as tables. The availability profiles of the studied pharmaceutical agents were presented as figures.

The determination of the cost estimate of diagnosing and managing PCa employed sensitivity analysis, which considered services provided to patients on request or for metastatic PCa as sensitivity factors. The median drug price ratio (MDPR) was calculated to determine the affordability of the PCa pharmacotherapeutic agents.

## Results

### Direct health cost estimates

#### Cost of prostate cancer diagnosis

The direct health cost estimates for diagnosing PCa are outlined in Table 1 and all details could be found in the table. The direct cost components include patient entry; screening laboratory investigations; and clinical management decision investigations. The researchers found that the public hospital charged patients for registration and consultation whilst the private hospital only charged patients a consultation approximately five times the bill incurred at the public facility. The results indicated that amongst the four private laboratories recruited for the study, only one charged their patients; usually for metastatic prostate cancers, an optional consultation of GHC 100.00 ($17.24). Therefore, for patients who required an external laboratory consultation, the total median modular service charge of GHC 96.00 ($16.55) increased by the stated sensitivity amount.

**Table 1:** Detailed price for the diagnosis of prostate cancer

Module/Service	Public Hospital GHC ($)	Private Hospital GHC ($)	Median Price (Private Laboratories) GHC ($)	Median Price (All Facilities) GHC ($) [Range, GHC]	Total Price Per Module (All Facilities) GHC ($)	Proportion of Total Cost (%)	% Change (per module)
**Patient Entry**							
Registration	11.00 (1.90)	–	–	11.00 (1.90) [11.00]	(−SF) = 96.00 (16.55) (+SF) = 196.00 (33.78)	(−SF) = 5.694 (+SF) = 2.850	− 2.844
Consultation	20.00 (3.45)	150.00 (25.85)	^b^100.00 (17.24)	85.00 (14.65) [20.00 - 150.00]			
**Screening Tests**							
PSA	80.00 (13.79)	80.00 (13.79)	80.00 (13.79)	80.00 (13.79) [50.00 - 100.00]	1205.00 (207.68)	(−SF) = 71.471 (+SF) = 17.525	−53.946
Free PSA	–	–	115.00 (19.82)	115.00 (19.82) [80.00 - 150.00]			
DRE	–	–	–	–			
TRUS	–	160.00 (27.58)	–	160.00 (27.58) [160.00]			
Biopsy (Histopathology)	750.00 (129.26)	850.00 (146.50)	950.00 (163.73)	850.00 (146.50) [750.00 - 950.00]			
**Clinical Management Decision Tests**							
Gleason grading on biopsies	150.00 (25.85)	150.00 (25.85)	250.00 (43.09)	150.00 (25.85) [150.00 - 250.00]	(−SF) = 385.00 (66.36)	(−SF) = 22.835 (+SF) = 79.625	+ 56.790
LFT	70.00	80.00 (13.79)	70.00 (12.06)	70.00 (12.06) [50.00 - 95.00]			
RFT	80.00 (13.79)	80.00 (13.79)	80.00 (13.79)	80.00 (13.79) [70.00 - 80.00]			
FBC	40.00 (6.89)	40.00 (6.89)	40.00 (6.89)	40.00 (6.89) [40.00 - 50.00]			
Blood Group	10.00 (1.72)	–	20.00 (3.45)	15.00 (2.59) [10.00 - 30.00]			
Serum phosphatases (acid/alkaline)	–	–	30.00 (5.17)	30.00 (5.17) [30.00 - 60.00]			
^a^CT Scan	–	–	800.00 (137.88)	800.00 (137.88) [800]	(+SF) = 5475.00 (943.62)		
^a^MRI Scan (Whole Body Diffusion)	–	–	4200.00 (723.88)	4200.00 (723.88) [4200.00]			
^a^Chest X-ray	80.00 (13.79)	–	95.00 (16.37)	90.00 (15.51) [80.00 - 100.00]			

*SF* Sensitivity Factor, *−SF* Exclusive of SF, *+SF* Inclusive of SF, *SMP-SF* Sum of Modular Prices Exclusive of SF, *SMP + SF* Sum of Modular Prices Inclusive of SF

^a^Component of Sensitivity Factor

^b^SF not added to the median price of all facilities (an optional service that is provided on request, usually in metastasis); SMP-SF = GHC 1686.00 ($ 290.58); SMP + SF = GHC 6876.00 ($ 1185.09); Modular Price Ratio = [(SMP + SF) ÷ (SMP-SF)] = 4.08

Amongst the 5 screening laboratory workouts: PSA, free PSA, digital rectal examination (DRE), TRUS, and biopsy, only the cost of PSA was the same at all the study sites (GHC 80.00: $ 13.79). The DRE offered by the hospitals did not attract any cost component. Again, the public hospital provided the cheapest biopsy services at the cost of GHC 750.00 ($129.26), whilst one private laboratory provided a biopsy service at GHC 950.00 ($ 163.73), the most expensive. Two private laboratories and a private hospital provided free PSA and USG services at GHC 115.00 ($ 26.71) median price and GHC 160.00 ($ 27.58). There were no optional services and hence, no sensitivity factor. The total median modular service charge was GHC 1205.00 ($ 207.68).

In estimating the modular price for the clinical management decision investigations, the researchers discovered that none of the hospitals was in the position to assess the serum phosphatases (acid/alkaline) of patients, perform a CT scan and an MRI scan (Whole Body Diffusion) at the time of the data collection. Also, the private hospital was not equipped to provide chest X-ray services and blood group determination. Amongst the four (4) private laboratories, one specialized in diagnostic imaging (CT scan and MRI), whilst the three (3) were equipped to provide all the blood work. Only one private laboratory provided the Gleason grading on biopsies service at a price of GHC 100.00 ($ 17.24) higher than the hospitals’ quote. We were informed that the imaging techniques (the CT scan, the MRI, and the chest X-ray) were done to investigate metastasis and hence, were classified as sensitivity factors. The median sum of these sensitivity factors was GHC 5475.00 ($ 943.62) compared to the median modular price without the diagnostic imaging, GHC 385.00 ($ 66.36). Hence, a metastatic PCa patient would spend GHC 5860.00 ($ 1009.98) to enable the multidisciplinary prostate cancer team to make a management decision.

From these results, we submit that for a metastatic PCa case requiring all clinical investigations and private laboratory consultation, the direct cost of diagnosis is estimated at GHC 6876.00 ($ 1185.09) compared to GHC 1686.00 ($ 290.58) for non-metastatic cases that do not require private laboratory consultation. Hence, metastatic prostate cancer increases the diagnostic cost by approximately 4 folds (Modular Price Ratio: 4.08) of non-metastatic prostate cancers.

#### Stage-specific management approaches and cost

According to the management protocols of the selected hospitals: Table 2, which are mainly influenced by the stage of the disease, the availability of equipment, and the goals of management, the cancer centre of the public hospital mainly managed PCa with Extended Beam Radiotherapy (EBRT) whilst the private hospital’s option was radical prostatectomy. For these two management arms, we found that patients who opted for radical prostatectomy paid GHC 2150.00 ($ 370.56) higher than patients who were managed on EBRT (GHC 2150.00: $ 370.56). These management options applied to localized PCa of low and moderate grades. However, the public hospital reported including a 6-month androgen deprivation therapy (ADT), at an additional cost of GHC 3000.00 ($ 517.05), for the management of moderate grade localized PCa depending on an assessment of beneficial outcome. The private facility managed locally advanced and metastatic PCa respectively with radiotherapy and ADT, and ADT alone. The radiotherapy needs of the patients being managed by the private hospital were met by the public hospital through a referral and follow-up. The allocation of cost components to these management options was not possible because officials were not privy to the financial commitments made by patients at the various radiotherapy referral centres in the country. Likewise, the cost of ADT for referred patients was not reported because the patients either procured their medicines from community pharmacies or were supplied by the receiving radiotherapy centre. The public facility managed locally advanced PCa with EBRT and ADT for 18 to 24 months. In managing metastatic PCa, the public facility could not allocate management costs because the patients had varied organ metastasis, and most of their prescriptions were obtained from community pharmacies that are walking distance from the hospital. However, the approach included ADT alone or ADT plus palliative radiotherapy or ADT plus abiraterone/docetaxel plus palliative radiotherapy. In instances where the patient had bone metastasis, the management included the addition of bisphosphonates. Goserelin was the ADT agent used by the two hospitals. Further details are contained in Table 2.

**Table 2:** Detailed stage-specific management price of prostate cancer

Invasive category	Gleason grade	TNM stage	Public Hospital		Private Hospital	
			First line management option	Unit price GHC [$]	First line management option	Unit price GHC [$]
Localized	6 Low	1, 2 A	Extended Beam Radiotherapy (EBRT) alone	12,950.00 [2231.95]	Radical prostatectomy	15,000.00 [2585.27]
	7 Moderate	2 B-C	EBRT (+ 6 months of Androgen Deprivation Therapy (ADT), depending on assessment of benefit outcome)	12,950.00 [2231.95] (+ 3000.00 [517.05])^a^	Radical prostatectomy	15,000.00 [2585.27]
Locally advanced	8,9,10 High	3 A-C	EBRT (+ 18-24 months ADT)	12,950.00 [2231.95] (+ 3000.00 [517.05] per 6 months)	Radiotherapy (from a referral facility) + ADT	“Missing”
Metastasis	8,9,10 High	4 A-B	ADT alone or ADT + palliative radiotherapy or ADT + palliative radiotherapy + abiraterone acetate/docetaxel All options plus bisphosphonate (for bone metastasis)	“Missing”	ADT alone	“Missing”

*EBRT* Extended Beam Radiotherapy, *ADT* Androgen Deprivation Therapy, *TNM* Tumour, Node, Metastasis; “Missing”: Unable to determine the cost

^a^Only paid when ADT is included

#### Availability and prices of pharmaceuticals in sampled community pharmacies

**Fig. 1:**
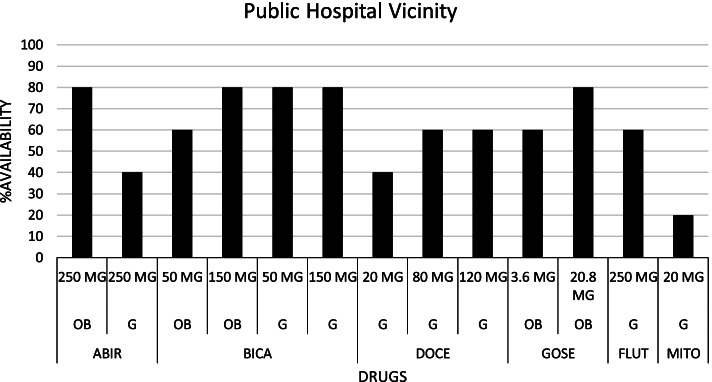
Availability pattern of Prostate Cancer drugs in Public Hospital Vicinity. [ABIR: Abiraterone acetate; BICA: Bicalutamide; DOCE: Docetaxel; FLUT: Flutamide; GOSE: Goserelin; MITO: Mitoxantrone; OB: Originator brand; G: Generic; MG: Milligram]

**Fig. 2:**
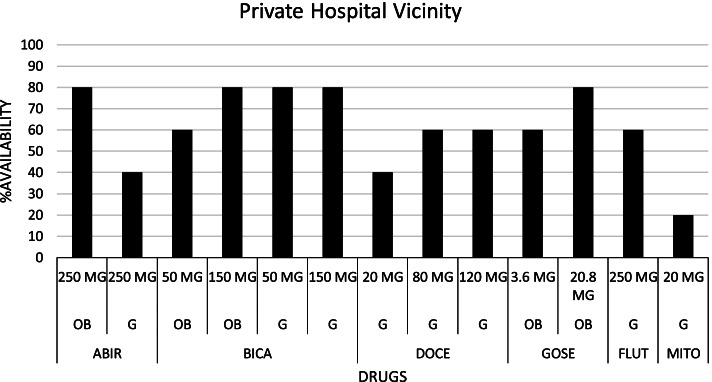
Availability pattern of Prostate Cancer drugs in Private Hospital Vicinity. [ABIR: Abiraterone acetate; BICA: Bicalutamide; DOCE: Docetaxel; FLUT: Flutamide; GOSE: Goserelin; MITO: Mitoxantrone; OB: Originator brand; G: Generic; MG: Milligram]

The availability profile and prices of the pharmaceutical agents listed by the recruited hospitals for the management of PCa are presented in Table 3. The drug list included abiraterone acetate (250 mg), bicalutamide (50 mg and 150 mg), docetaxel (20 mg, 80 mg and 120 mg), flutamide (250 mg), goserelin (3.6 mg and 10.8 mg) and mitoxantrone (20 mg). We found that all the drugs with the indicated strengths were available in community pharmacies located within the vicinity of the public hospital: Fig. 1. Within the vicinity of the private hospital: Fig. 2, we noticed that none of the pharmacies could supply generics of abiraterone acetate (250 mg), docetaxel (80 mg), and mitoxantrone (20 mg), and only one pharmacy could supply generics of docetaxel (20 mg and 120 mg). Also, only two community pharmacies had the originator brand of abiraterone acetate (250 mg). The mean availability of all the drugs in the community pharmacies located within the vicinity of the public hospital, private hospital, and the combination of the two sites were respectively 61.54, 41.54, and 51.54%. Therefore, none of the drugs had absolute availability at the ten community pharmacies that were sampled. Details of the results are presented in Table 3.

**Table 3:** Price and availability details of prostate cancer pharmaceuticals in sampled community pharmacies

SN	Prostate cancer medication	Originator brand (OB) or Generic (G)	Strength	Dosage form/unit	Pack size	Unit MLCP GHC ($) [Range, GHC]	Unit MIRP GHC ($)	MDPR	% Availability (***n*** = 10)
1	Abiraterone acetate	OB	250 mg^a^	Tablet	120	39.42 (6.79) [33.75 - 65.70]			60.00
		G	250 mg^a^	Tablet	120	25.00 (4.31) [20.83 - 29.16]			20.00
2	Bicalutamide	OB	50 mg	Tablet	28	9.98 (1.72) [8.60 - 10.36]	1.36 (0.23)	7.34	60.00
		OB	150 mg	Tablet	28	27.60 (4.76) [21.07 - 32.86]	5.98 (1.03)	4.62	90.00
		G	50 mg	Tablet	28	3.00 (0.52) [1.80 - 6.90]	1.36 (0.23)	2.21	70.00
		G	150 mg	Tablet	28	4.29 (0.74) [3.05 - 8.40]	5.98 (1.03)	0.72	70.00
3	Docetaxel	G	20 mg	Injection	1	280.00 (48.26) [90.00 - 300.00]	235.10 (40.52)	1.19	30.00
		G	80 mg	Injection	1	400.00 (68.94) [350.00 - 560.00]	278.46 (47.99)	1.44	30.00
		G	120 mg^a^	Injection	1	750.00 (129.26) [420.00 - 1200.00]			40.00
4	Flutamide	G	250 mg	Tablet	84	10.00 (1.72) [3.50 - 18.25]	0.65 (0.11)	15.38	50.00
5	Goserelin	OB	3.6 mg^a^	Injection	1	662.50 (114.18) [530.00 - 689.00]			70.00
		OB	10.8 mg^a^	Injection	1	1700.00 (293.00) [1509.70 - 1958.80]			70.00
6	Mitoxantrone	G	20 mg^a^	Injection	1	205.00 (35.33) [250]			10.00

MDPR = (MLCP ÷ MIRP); Mean Drug Availability (All Pharmacies) = 51.54%; Mean Drug Availability (Private Hospital Vicinity) = 41.54%; Mean Drug Availability (Public Hospital Vicinity) = 61.54%

*MLCP* Median Local Consumer Price, *MIRP* Median International Reference Price, *MDPR* Median Drug Price Ratio

^a^Drugs/Drug strengths without MIRP

The unit prices of the pharmaceutical agents have been quoted in Table 3. The median local consumer price (MLCP) per unit dose of the investigated drugs has been collated and presented with the unit median international reference price (MIRP). The median drug price ratio (MDPR) of the medications was determined to be in the range of 0.72 and 15.38. The MDPR could not be calculated for abiraterone acetate, docetaxel (120 mg), goserelin, and mitoxantrone due to the unavailability of the MIRP. The originator brand of bicalutamide and generic flutamide had MDPR greater than 4.00 with the flutamide recording the highest (15.38). Comparing the MDPR of bicalutamide (50 mg) to bicalutamide (150 mg), we discovered the former was 2.72 higher than the latter.

## Discussion

Our study investigated the direct cost incurred by patients and caregivers in diagnosing and managing prostate cancer in Ghana, using the Ashanti Region as the study site. Although the researchers relied on the prevalence approach in the cost-of-illness study, the absence of a national cancer financing agency and a robust database with evidence of the financial commitment of patients resulted in the sourcing of data from healthcare facilities. We discovered that patients paid for facility entry which comprised patient registration and consultancy services. These services cost all patients a median of GHC 96.00 ($ 16.55), with metastatic prostate cancer cases costing an additional GHC 100.00 ($ 17.24) for specialized consultancy services. In other studies, the direct cost estimate was not restricted to management cost but other cost components that included administrative charges where higher cost was associated with metastatic cases [23, 24]. In our study, the higher cost was linked to the source of care where consultancy service from the private facility was approximately five times the sum of registration and consultancy fees from the public hospital.

In determining the cost of diagnosing PCa, we investigated other cost modules, including screening laboratory investigations and clinical management decision investigations. For the screening laboratory investigations, the researchers found that all patient categories paid a median of GHC 1205.00 ($ 207.68). From the breakdown of this module, the private and public hospitals did not charge patients for DRE. However, pathological investigation costs patients a median of GHC 850.00 ($ 146.50). Again, the privatization of this service increased cost because the public hospital charged GHC 100.00 ($ 17.24) lesser than the median cost whilst the private laboratories charged GHC 100.00 ($ 17.24) above the median cost. This finding signals possible over-pricing by private healthcare providers and hence, an increased cost burden which would eventually reduce the quality of care and increase mortality [25, 26]. Clinical management decision investigations contributed to an additional median cost of GHC 385.00 ($ 66.36) for all categories of patients with some specialized investigations required for metastatic cases attracting an extra cost of GHC 5475.00 ($ 943.62). This resonates with several studies that have confirmed an increased cost-of-illness associated with metastatic cancers [9, 27]. Although service privatization increased cost, this phenomenon increased service availability [26], and the patients did not have to travel outside the Ashanti Region to source these services.

Our study findings have respectively established the diagnosis of metastatic and non-metastatic PCa at a median cost of GHC 6876.00 ($ 1185.09) and GHC 1686.00 ($ 290.58). Although patient entry cost for non-metastatic PCa contributed to a proportional diagnostic cost of 5.694%, this was reduced to 2.850% for metastatic PCa. Screening laboratory investigations for non-metastatic and metastatic PCa respectively contributed to proportional diagnostic costs of 71.471 and 17.525%, whilst proportional diagnostic cost for clinical management decision investigations for non-metastatic PCa at 22.835% increased to 79.625% with metastasis. These findings confirm the increased economic burden of PCa with metastasis and the need for early detection [6]. A cost analysis of the stage-specific management approaches of PCa revealed radical prostatectomy and Extended Beam Radiotherapy (EBRT) as the mainstays in the management of localized PCa [28] at GHC 15,000.00 ($ 2585.27) and GHC 12,950.00 ($ 2231.95), respectively. The researchers found that disease progression increased cost-of-illness relying on the difficulty in the cost estimation of the management of locally advanced and metastatic PCa [6]. The phenomenon of cost accumulation was observed with disease progression resulting from the increased utilization of expensive pharmaceutical agents [24].

An assessment of the availability of the master list of pharmaceutical agents was done to determine patients’ access to these agents without a focus on the cost of transportation because the pharmacies were within walking distance of the hospitals. We observed that the community pharmacies within the vicinity of the public hospital had a better availability profile than the private hospital. This could be due to the lower diagnostic and management costs associated with seeking care from the public hospital and the introduction of pharmaceutical agents in the management plan of localized PCa compared to the private hospital. The former reason could have attracted more patients to the public hospital and hence, the latter reason serves as a competition motivator between the community pharmacies to meet the pharmaceutical demands of these patients. It is important to stress the effect of the Coronavirus disease (COVID-19) on drug availability in resource constraint countries, including Ghana [29, 30]. Therefore, the researchers, having conducted the study in the heat of the COVID-19 pandemic, ascribe the availability profile of the sampled drugs to the pandemic. This phenomenon is proposed to have affected the price and affordability of these medications.

The price and affordability of the surveyed pharmacotherapeutic agents were determined using the 2015 Edition of the International Medical Products Price Guide (IMPPG) [22], the 2nd August 2021 foreign exchange rate of the Bank of Ghana (USD 1.00: GHC 5.8021), and the 2021 Ghana National Daily Minimum Wage (GNDMW) of GHC 12.53. According to the calculated MDPR of the medications whose unit MIRP was listed in the IMPPG, the MDPR ranged from 0.72 to 15.38 with generic flutamide as the most expensive (15.38) [31]. Also, the MDPR of all the available strengths of the originator brand of bicalutamide was greater than 4.00 and hence, a reflection of poor affordability [31]. The affordability challenge of bicalutamide was addressed (MDPR less than 4) with the availability profile of the generic (70% availability). The MDPR of bicalutamide (50 mg) was 2.73 higher than bicalutamide (150 mg). Although most drugs had an MDPR of less than 4, a reflection of affordability, the average Ghanaian disagrees based on a minimum daily wage of GHC 12.53 ($ 0.46) [32].

### Implications for prostate cancer care, financing, and research

From the study findings, we acknowledge the unfavorable financial burden of PCa on Ghanaians. This high direct health cost of PCa, as found in our study, could be attributed to the absence of subsidies to private healthcare providers. The researchers did not come across any Ghanaian study that examined the cost of PCa. Therefore, it has become imperative to conduct more studies to highlight the cost of the disease, not limited to the direct health cost, and make recommendations to improve PCa care in Ghana. Finally, we propose that further research must be conducted to evaluate the effect of this unfavorable COI parameter on prostate cancer management outcomes.

### Strengths and limitations

This Ghanaian study examined the direct health cost of prostate cancer on patients and caregivers by reporting on the direct diagnostic and management cost. Although we managed to inform the scientific community about the direct health cost of the disease, we limited the study to the Ashanti Region of Ghana. This affects the generalizability of the findings since the study site does not represent the holistic happenings in Ghana. In the absence of data, we could not study the whole spectrum of COI. However, in the absence of similar studies, we present this as a representation of the Ghanaian situation and recommend a PCa COI study using the incidence approach. Other limitations of the study include the exclusion of medications such as ketoconazole (oral) and bisphosphonates (oral) on grounds that there are no classical chemotherapeutic agents. Also, palliative and pain medications were not factored in the cost estimation of management. The lack of the median international reference prices of 3 out of the 6 medications studied did not permit the assessment of the affordability of these affected medications. We cannot ignore the effect of COVID-19 on the drug availability pattern and the cost. For pharmacies that did not have some drugs at the time of the survey, they probably would have had them at different times of the year. Hence, the drug availability pattern is affected by the time of the survey.

## Recommendation and conclusion

Cost-of-Illness studies inform policy-making regarding health economics. The direct cost of prostate cancer diagnosis and management was studied with various challenges due to the paucity of data. Although a full assessment of the facets of COI was not feasible, the paper successfully highlighted the direct health cost of the disease. With this evidence, it has become imperative for policy-makers, Governmental agencies and non-Governmental agencies to be involved in addressing the economic burden of prostate cancer in Ghana.

## Supplementary Information

**Additional file 1.** Prostate cancer diagnosis cost sheet

**Additional file 2.** Prostate cancer stage-specific management cost sheet

**Additional file 3.** The availability and prices of prostate cancer drugs

## Data Availability

The datasets generated and/or analyzed during the current study are not publicly available due to institutional/study sites’ trade secrets but are available from the corresponding author on reasonable request.

## Abbreviations

ADT: Androgen-deprivation Therapy
COI: Cost-of-Illness
COVID-19: Coronavirus Disease
CT: Computed Tomography
DRE: Digital Rectal Examination
EBRT: Extended Beam Radiotherapy
FBC: Full Blood Count
GHC: Ghana Cedi
GLOBOCAN: Global Cancer Incidence, Mortality and Prevalence
GNDMW: Ghana National Daily Minimum Wage
IMPPG: International Medical Products Price Guide
KBTH: Korle-Bu Teaching Hospital
LFT: Liver Function Test
MDPR: Median Drug Price Ratio
MIRP: Median International Reference Price
MLCP: Median Local Consumer Price
MRI: Magnetic Resonance Imaging
NHIS: National Health Insurance Scheme
TNM: Tumour, Node, Metastasis
TRUS: Transrectal Ultrasound
PCa: Prostate cancer
PI: Principal Investigator
PSA: Prostate-specific Antigen
RFT: Renal Function Test
SF: Sensitivity Factor
SMP: Sum of Modular Prices
USD: United States Dollar
X-ray: Radiograph
