# Correction: The direct cost incurred by patients and caregivers in diagnosing and managing prostate cancer in Ghana

**DOI:** 10.1186/s12913-022-08535-9

**Published:** 2022-09-14

**Authors:** Ebenezer Wiafe, Kof Boamah Mensah, Kwaku Addai Arhin Appiah, Frasia Oosthuizen, Varsha Bangalee

**Affiliations:** 1 Discipline of Pharmaceutical Sciences, College of Health Sciences, University of KwaZulu-Natal, Durban, South Africa; 2 Clinical Pharmacy Services Unit, Directorate of Pharmacy, Ho Teaching Hospital, Ho, Ghana; 3 Department of Pharmacy Practice, Faculty of Pharmacy and Pharmaceutical Sciences, College of Health Sciences, Kwame Nkrumah University of Science and Technology, Kumasi, Ghana; 4 Department of Surgery, School of Medicine and Dentistry, College of Health Sciences, Kwame Nkrumah University of Science and Technology, Kumasi, Ghana

**Correction: BMC Health Serv Res 22, 1105 (2022)**

**https://doi.org/10.1186/s12913-022-08476-3**

Following publication of the original article [1], the authors identified that a duplicated version of Fig. 1 was published as Fig. 2. The correct Fig. 2 is included in this Correction and the original article has been corrected.

**Fig. 2:**
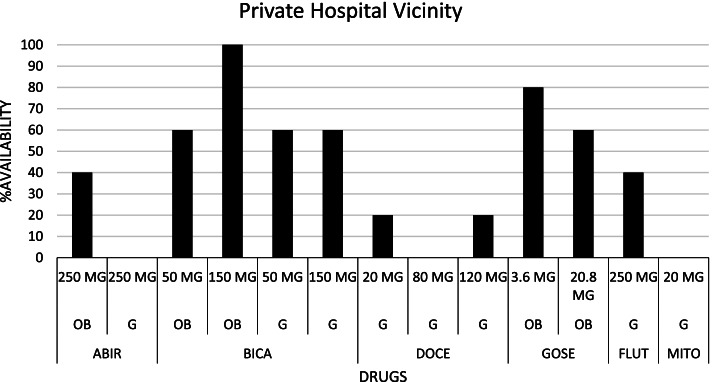
Availability pattern of Prostate Cancer drugs in Private Hospital Vicinity. [ABIR: Abiraterone acetate; BICA: Bicalutamide; DOCE: Docetaxel; FLUT: Flutamide; GOSE: Goserelin; MITO: Mitoxantrone; OB: Originator brand; G: Generic; MG: Milligram]
