# Supplementary material for: The direct cost incurred by patients and caregivers in diagnosing and managing prostate cancer in Ghana
Source: BMC Health Serv Res. 2022 Aug 31;22:1105. doi: 10.1186/s12913-022-08476-3 (PMC9428865; doi:10.1186/s12913-022-08476-3)
Supplement: Supplementary file 1 — Additional file 1. Prostate cancer diagnosis cost sheet [file 12913_2022_8476_MOESM1_ESM.docx]

**The direct cost incurred by patients and caregivers in diagnosing and managing prostate cancer in Ghana.**

**ADDITIONAL FILE 1: PROSTATE CANCER DIAGNOSIS COST SHEET**

**FACILITY CODE: …………………….**

1. **PATIENT ENTRY COST**

| **SN** | **ITEM** | **UNIT COST (GHC)** |
| --- | --- | --- |
| 1 | Registration |  |
| 2 | Consultation |  |
| 3 | Other(s). Please specify: |  |

1. **SCREENING TESTS**

| **SN** | **ITEM** | **UNIT COST (GHC)** |
| --- | --- | --- |
| 1 | PSA |  |
| 2 | Free PSA |  |
| 3 | DRE |  |
| 4 | TRUS |  |
| 5 | Biopsy (Histopathology) |  |

1. **CLINICAL MANAGEMENT DECISION TESTS**

| **SN** | **ITEM** | **UNIT COST (GHC)** |
| --- | --- | --- |
| 1 | Gleason grading on biopsies |  |
| 2 | LFT |  |
| 3 | RFT |  |
| 4 | FBC |  |
| 5 | Blood Group |  |
| 6 | Serum phosphatases (acid/alkaline) |  |
| 7 | CT Scan |  |
| 8 | MRI Scan (Whole Body Diffusion) |  |
| 9 | Chest X-ray |  |

For hospitals, kindly list private laboratories or diagnostic/imaging establishments that support you to provide the above services:

1.

2.

3.
