# Supplementary material for: The direct cost incurred by patients and caregivers in diagnosing and managing prostate cancer in Ghana
Source: BMC Health Serv Res. 2022 Aug 31;22:1105. doi: 10.1186/s12913-022-08476-3 (PMC9428865; doi:10.1186/s12913-022-08476-3)
Supplement: Supplementary file 2 — Additional file 2. Prostate cancer stage-specific management cost sheet [file 12913_2022_8476_MOESM2_ESM.docx]

**The direct cost incurred by patients and caregivers in diagnosing and managing prostate cancer in Ghana.**

**ADDITIONAL FILE 2: PROSTATE CANCER STAGE-SPECIFIC MANAGEMENT COST SHEET**

**FACILITY CODE: …………………….**

| **INVASIVE CATEGORY** | **GLEASON GRADE** | **TNM STAGE** | **MANAGEMENT** | | **ONCOLOGY PHARMACEUTICAL AGENTS EMPLOYED IN PROSTATE CANCER MANAGEMENT** |
| --- | --- | --- | --- | --- | --- |
|  |  |  | **FIRST LINE** | **COST (GHC)** |  |
| Localized | 6  Low | 1,2A |  |  | 1.  2.  3.  4.  5.  6.  7.  8.  9.  10. |
|  | 7  Moderate | 2B-C |  |  |  |
| Locally advanced | 8,9,10  High | 3A-C |  |  |  |
| Metastasis | 8,9,10  High | 4A-B |  |  |  |
