# Supplementary material for: The direct cost incurred by patients and caregivers in diagnosing and managing prostate cancer in Ghana
Source: BMC Health Serv Res. 2022 Aug 31;22:1105. doi: 10.1186/s12913-022-08476-3 (PMC9428865; doi:10.1186/s12913-022-08476-3)
Supplement: Supplementary file 3 — Additional file 3. The availability and prices of prostate cancer drugs [file 12913_2022_8476_MOESM3_ESM.docx]

**The direct cost incurred by patients and caregivers in diagnosing and managing prostate cancer in Ghana.**

**ADDITIONAL FILE 3: THE AVAILABILITY AND PRICES OF PROSTATE CANCER DRUGS**

**FACILITY CODE: …………………….**

| **SN** | **PROSTATE CANCER MEDICATION** | **ORIGINATOR BRAND (OB) OR GENERIC (G)** | **STRENGTH** | **DOSAGE FORM/UNIT** | **PACK SIZE** | **UNIT COST (GHC)** |
| --- | --- | --- | --- | --- | --- | --- |
| 1 | Abiraterone acetate |  |  |  |  |  |
|  |  |  |  |  |  |  |
| 2 | Bicalutamide |  |  |  |  |  |
|  |  |  |  |  |  |  |
| 3 | Docetaxel |  |  |  |  |  |
|  |  |  |  |  |  |  |
| 4 | Flutamide |  |  |  |  |  |
|  |  |  |  |  |  |  |
| 5 | Goserelin |  |  |  |  |  |
|  |  |  |  |  |  |  |
| 6 | Mitoxantrone |  |  |  |  |  |
|  |  |  |  |  |  |  |
